# Tris(4-formyl­phen­yl)phosphane oxide tetra­hydro­furan hemisolvate

**DOI:** 10.1107/S1600536813020059

**Published:** 2013-07-31

**Authors:** James Kakoullis, Frank R. Fronczek, Andrew W. Maverick

**Affiliations:** a Department of Chemistry, Louisiana State University, Baton Rouge, LA 70803-1804, USA

## Abstract

The title compound, C_21_H_15_O_4_P·0.5C_4_H_8_O, contains an ordered phosphane oxide in a general position and a tetra­hydro­furan solvent mol­ecule disordered about a twofold axis. All three aldehyde substituents are nearly coplanar with their attached benzene rings, with C—C—C—O torsion angles in the range 1.64 (17)–4.24 (19)°. All three have different conformations with respect to the P=O group, one *syn*, one *anti*, and one *gauche*. Two of the aldehyde substituents form inter­molecular C—H⋯O contacts.

## Related literature

For synthetic procedures, see: Bartlett *et al.* (1978 ▶); Chalier *et al.* (1996 ▶); Kumagai & Itsuno (2001 ▶). For use as a precursor in supra­molecular chemistry, see: Kakoullis (2007 ▶); Pariya *et al.* (2008 ▶). For weak hydrogen bonds, see: Desiraju & Steiner (1999 ▶). For related structures, see: Daly (1964 ▶); Etter & Baures (1988 ▶); Siegler *et al.* (2007 ▶); Spek (1987 ▶); Brock *et al.* (1985 ▶); Lenstra (2007 ▶); Thierbach *et al.* (1980 ▶); Baures & Silverton (1990 ▶); Baures (1991 ▶).
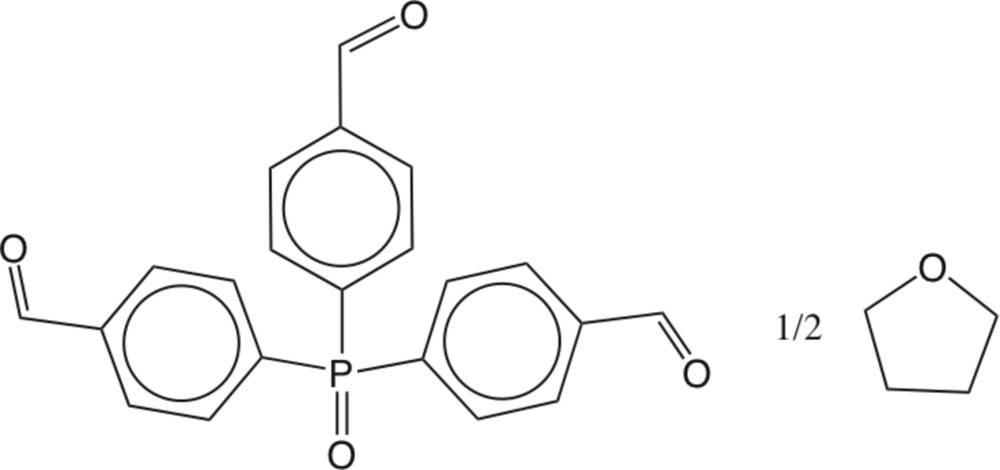


## Experimental

### 

#### Crystal data

C_21_H_15_O_4_P·0.5C_4_H_8_O
*M* *_r_* = 398.35
Monoclinic, 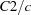

*a* = 21.371 (3) Å
*b* = 13.474 (2) Å
*c* = 13.436 (2) Å
β = 99.018 (9)°
*V* = 3821.1 (10) Å^3^
*Z* = 8
Mo *K*α radiation
μ = 0.17 mm^−1^
*T* = 110 K
0.45 × 0.43 × 0.38 mm

#### Data collection

Nonius KappaCCD diffractometer
Absorption correction: multi-scan (*SCALEPACK*; Otwinowski & Minor, 1997 ▶) *T*_min_ = 0.926, *T*_max_ = 0.937
36155 measured reflections
7598 independent reflections
5928 reflections with *I* > 2σ(*I*)
*R*_int_ = 0.023

#### Refinement

*R*[*F* ^2^ > 2σ(*F* ^2^)] = 0.042
*wR*(*F* ^2^) = 0.116
*S* = 1.03
7598 reflections
280 parameters
H-atom parameters constrained
Δρ_max_ = 0.40 e Å^−3^
Δρ_min_ = −0.39 e Å^−3^

### 

Data collection: *COLLECT* (Nonius, 2000 ▶); cell refinement: *SCALEPACK* (Otwinowski & Minor, 1997 ▶); data reduction: *DENZO* (Otwinowski & Minor, 1997 ▶) and *SCALEPACK*; program(s) used to solve structure: *SIR97* (Altomare *et al.*, 1999 ▶); program(s) used to refine structure: *SHELXL97* (Sheldrick, 2008 ▶); molecular graphics: *ORTEP-3 for Windows* (Farrugia, 2012 ▶); software used to prepare material for publication: *SHELXL97*.

## Supplementary Material

Crystal structure: contains datablock(s) global, I. DOI: 10.1107/S1600536813020059/fj2638sup1.cif

Structure factors: contains datablock(s) I. DOI: 10.1107/S1600536813020059/fj2638Isup2.hkl

Supplementary material file. DOI: 10.1107/S1600536813020059/fj2638Isup3.cml

Additional supplementary materials:  crystallographic information; 3D view; checkCIF report

## Figures and Tables

**Table 1:** Hydrogen-bond geometry (Å, °)

*D*—H⋯*A*	*D*—H	H⋯*A*	*D*⋯*A*	*D*—H⋯*A*
C7—H7⋯O3^i^	0.95	2.56	3.4303 (16)	152
C14—H14⋯O1^ii^	0.95	2.50	3.1575 (14)	127

Symmetry codes: (i) 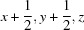; (ii) 
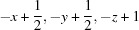
.

**Fig. 1.**
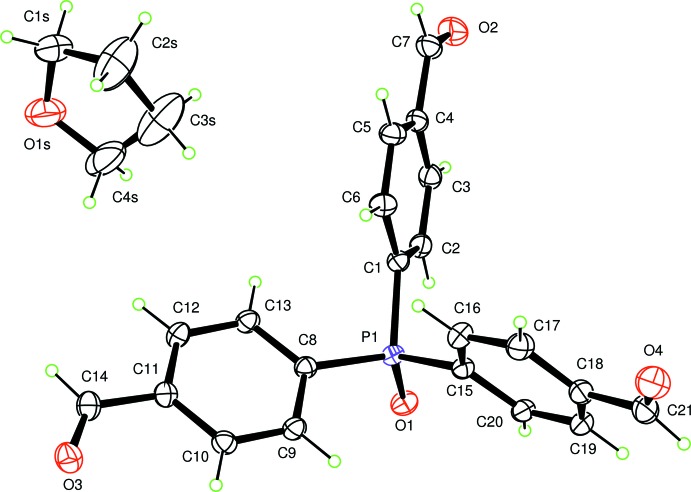
Numbering scheme and ellipsoids at the 50% level. H atoms are represented with arbitrary radius. Only one orientation of the disordered solvent molecule is shown.

## supplementary crystallographic information

### Comment

Triphenylphosphane oxide (TPPO) has been extensively structurally studied, as a result of the high basicity of its O atom, which makes it an excellent hydrogen-bond acceptor. Its utility as a crystallization aid for molecules having hydrogen-bond donors was reported by Etter & Baures (1988), which has led to its use in forming molecular cocrystals (Siegler *et al.*, 2007). Also, it has four known polymorphs (Spek, 1987; Brock *et al.*, 1985; Lenstra, 2007) and several known solvates (Thierbach *et al.*, 1980; Baures & Silverton, 1990; Baures, 1991).

Tris(4-formylphenyl)phosphane was first reported by Bartlett *et al.* (1978), but was also synthesized later by other groups (Chalier *et al.*, 1996). Our interest in the phosphane and the corresponding phosphane oxide (I) stems from their use as precursors to multifunctional ligands for supramolecular chemistry (Kakoullis, 2007; Pariya *et al.*, 2008;). The structure of (I) is illustrated in Fig. 1, showing only one orientation of the THF solvent molecule, which is disordered about a twofold axis. Atom C2S lies 0.51 Å from that axis, and the nearest distance between partially populated sites is 1.00 Å (C2S···C3S at 1 - *x*, *y*, 3/2 - *z*), so the resolution of the data (0.64 Å) allowed individual refinement of the partially populated positions without constraint. The three aldehyde substituents are nearly coplanar with the phenyl groups to which they are attached, with maximum torsion angle magnitude 4.24 (19) ° for C17—C18—C21—O4. The three 4-formylphenyl groups all have different conformations with respect to the phosphane oxide bond. Aldehyde C7═O2 is *syn* to P1═O1, with O1—P1···C7—O2 torsion angle -11.6 (1)°, while the corresponding torsion angles are 78.8 (1)° for O3 and -172.6 (1)° for O4. Intermolecular interactions include C—H···O contacts (Desiraju & Steiner, 1999) for two of the three aldehydes as donors. These are C7—H···O3(1/2 + *x*, 1/2 + *y*, *z*) with C···O distance 3.4303 (16) Å and 152° angle about H, and the shorter but less linear contact C14—H···O1(1/2 - *x*, 1/2 - *y*, 1 - *z*), 3.1575 (14) Å and 127°.

### Experimental

To prepare (I), the precursor tris(4-formylphenyl)phosphane was first prepared following and combining elements of the procedures for the synthesis of tris(4-formylphenyl)phosphane (Bartlett *et al.*, 1978) and bis(4-formylphenyl)dimethylsilane (Kumagai & Itsuno, 2001). A sample of 4-bromobenzaldehyde dimethyl acetal (5 ml, 29.9 mmol) was combined with 40 ml dry THF in an inert atmosphere in a round-bottom flask. The solution was brought to -78 °C under streaming N_2_, and n-butyllithium/hexanes 1.6 *M* (19.8 ml, 31.7 mmol) was added over approximately 1 h while stirring. The solution initially turned from colorless to light yellow, then to milky white. After 2 h, at -78 °C, PCl_3_ (0.80 ml, 9.17 mmol) was added over a period of 15 minutes. When the PCl_3_ was added, the solution turned orange-red. The solution was kept at -78 °C for another 1 h. Then the solution was allowed to come to room temperature over 1 h. The solvent was evaporated, leaving the crude acetal, which was dissolved in a mixture of dichloromethane and water. The solution was then washed: first with concentrated NaHCO_3_ and then with brine. The organic phase was dried over Na_2_SO_4_. The organic phase was then evaporated, leaving a residue (5.24 g). The crude material was dissolved in 50 ml THF and 50 ml 2 *M* HCl. The solution was stirred under reflux conditions for 1 h under a stream of N_2_. To the solution, 50 ml of water and 50 ml of ethyl acetate were added. The organic phase was then washed, first with concentrated NaHCO_3_ and then with brine, dried over Na_2_SO_4_ and evaporated, leaving a residue (3.97 g). This residue, which is crude tris(4-formylphenyl)phosphane and tris(4-formylphenyl)phosphane oxide (I), was dissolved in 25% CHCl_3_/ 75% ethyl acetate and applied to a silica gel column with 25% CHCl_3_/ 75% ethyl acetate as the mobile phase. The column was run as a flash column. This process yielded pure tris(4-formylphenyl)phosphane, 1.51 g, 44% yield. Continuing to run the flash column produced pure tris(4-formylphenyl)phosphane oxide (I),1.92 g, 56% yield. Crystals of (I) were prepared by evaporation of a solution in THF over one week.

### Refinement

H atoms were placed in idealized positions with C—H distances 0.95 - 0.99 Å and thereafter treated as riding. *U*_iso_ for H was assigned as 1.2 times *U*_eq_ of the attached C atoms. The THF molecule is disordered about a twofold axis, and its atoms were assigned half occupancy.

### Crystal data

**Table:**

C_21_H_15_O_4_P·0.5C_4_H_8_O	*F*(000) = 1664
*M**_r_* = 398.35	*D*_x_ = 1.385 Mg m^−^^3^
Monoclinic, *C*2/*c*	Mo *K*α radiation, λ = 0.71073 Å
Hall symbol: -C 2yc	Cell parameters from 7492 reflections
*a* = 21.371 (3) Å	θ = 2.5–33.7°
*b* = 13.474 (2) Å	µ = 0.17 mm^−^^1^
*c* = 13.436 (2) Å	*T* = 110 K
β = 99.018 (9)°	Fragment, yellow
*V* = 3821.1 (10) Å^3^	0.45 × 0.43 × 0.38 mm
*Z* = 8	

### Data collection

**Table:**

Nonius KappaCCD diffractometer	7598 independent reflections
Radiation source: fine-focus sealed tube	5928 reflections with *I* > 2σ(*I*)
Graphite monochromator	*R*_int_ = 0.023
ω and φ scans	θ_max_ = 33.7°, θ_min_ = 3.0°
Absorption correction: multi-scan (*SCALEPACK*; Otwinowski & Minor, 1997)	*h* = −33→33
*T*_min_ = 0.926, *T*_max_ = 0.937	*k* = −19→21
36155 measured reflections	*l* = −20→20

### Refinement

**Table:**

Refinement on *F*^2^	Primary atom site location: structure-invariant direct methods
Least-squares matrix: full	Secondary atom site location: difference Fourier map
*R*[*F*^2^ > 2σ(*F*^2^)] = 0.042	Hydrogen site location: inferred from neighbouring sites
*wR*(*F*^2^) = 0.116	H-atom parameters constrained
*S* = 1.03	*w* = 1/[σ^2^(*F*_o_^2^) + (0.0523*P*)^2^ + 2.5619*P*] where *P* = (*F*_o_^2^ + 2*F*_c_^2^)/3
7598 reflections	(Δ/σ)_max_ = 0.001
280 parameters	Δρ_max_ = 0.40 e Å^−^^3^
0 restraints	Δρ_min_ = −0.39 e Å^−^^3^

### Special details

**Table:**

Geometry. All e.s.d.'s (except the e.s.d. in the dihedral angle between two l.s. planes) are estimated using the full covariance matrix. The cell e.s.d.'s are taken into account individually in the estimation of e.s.d.'s in distances, angles and torsion angles; correlations between e.s.d.'s in cell parameters are only used when they are defined by crystal symmetry. An approximate (isotropic) treatment of cell e.s.d.'s is used for estimating e.s.d.'s involving l.s. planes.
Refinement. Refinement of *F*^2^ against ALL reflections. The weighted *R*-factor *wR* and goodness of fit *S* are based on *F*^2^, conventional *R*-factors *R* are based on *F*, with *F* set to zero for negative *F*^2^. The threshold expression of *F*^2^ > σ(*F*^2^) is used only for calculating *R*-factors(gt) *etc*. and is not relevant to the choice of reflections for refinement. *R*-factors based on *F*^2^ are statistically about twice as large as those based on *F*, and *R*- factors based on ALL data will be even larger.

### Fractional atomic coordinates and isotropic or equivalent isotropic displacement parameters (Å^2^)

**Table:**

	*x*	*y*	*z*	*U*_iso_*/*U*_eq_	Occ. (<1)
P1	0.328736 (12)	0.285691 (19)	0.269566 (19)	0.01652 (7)	
O1	0.27730 (4)	0.35710 (6)	0.23209 (6)	0.02304 (16)	
O2	0.58557 (5)	0.58191 (7)	0.41717 (7)	0.0341 (2)	
O3	0.21889 (4)	−0.06198 (6)	0.57196 (7)	0.02736 (18)	
O4	0.42853 (5)	−0.02731 (8)	−0.07657 (8)	0.0385 (2)	
C1	0.40140 (5)	0.34656 (8)	0.32453 (7)	0.01777 (18)	
C2	0.40518 (5)	0.44958 (8)	0.31497 (8)	0.02015 (19)	
H2	0.3691	0.4861	0.2848	0.024*	
C3	0.46164 (5)	0.49869 (8)	0.34949 (8)	0.0224 (2)	
H3	0.4643	0.5687	0.3425	0.027*	
C4	0.51435 (5)	0.44465 (8)	0.39446 (8)	0.02102 (19)	
C5	0.51021 (5)	0.34239 (9)	0.40684 (8)	0.0227 (2)	
H5	0.5459	0.3063	0.4393	0.027*	
C6	0.45398 (5)	0.29303 (8)	0.37179 (8)	0.0217 (2)	
H6	0.4512	0.2232	0.3798	0.026*	
C7	0.57583 (6)	0.49462 (10)	0.42927 (9)	0.0268 (2)	
H7	0.6096	0.4556	0.4632	0.032*	
C8	0.30889 (5)	0.20142 (7)	0.36429 (7)	0.01710 (17)	
C9	0.26866 (5)	0.12078 (8)	0.33428 (8)	0.01918 (19)	
H9	0.2543	0.1092	0.2647	0.023*	
C10	0.24976 (5)	0.05794 (8)	0.40579 (8)	0.01975 (19)	
H10	0.2230	0.0029	0.3855	0.024*	
C11	0.27051 (5)	0.07641 (8)	0.50828 (8)	0.01879 (18)	
C12	0.30915 (5)	0.15758 (8)	0.53806 (8)	0.0211 (2)	
H12	0.3223	0.1703	0.6078	0.025*	
C13	0.32874 (5)	0.22025 (8)	0.46671 (8)	0.01944 (18)	
H13	0.3554	0.2753	0.4873	0.023*	
C14	0.25276 (5)	0.01018 (8)	0.58758 (8)	0.0222 (2)	
H14	0.2692	0.0255	0.6557	0.027*	
C15	0.35007 (5)	0.21019 (7)	0.16835 (7)	0.01758 (18)	
C16	0.39073 (5)	0.12789 (8)	0.18493 (8)	0.0216 (2)	
H16	0.4074	0.1081	0.2517	0.026*	
C17	0.40635 (6)	0.07555 (9)	0.10328 (9)	0.0244 (2)	
H17	0.4348	0.0211	0.1141	0.029*	
C18	0.38019 (5)	0.10291 (9)	0.00515 (8)	0.0234 (2)	
C19	0.34019 (5)	0.18443 (9)	−0.01138 (8)	0.0236 (2)	
H19	0.3228	0.2031	−0.0782	0.028*	
C20	0.32550 (5)	0.23890 (8)	0.07013 (8)	0.02047 (19)	
H20	0.2988	0.2955	0.0588	0.025*	
C21	0.39321 (6)	0.04313 (10)	−0.08199 (9)	0.0303 (3)	
H21	0.3722	0.0616	−0.1469	0.036*	
O1S	0.45041 (11)	0.23429 (19)	0.84831 (19)	0.0494 (6)	0.50
C1S	0.51269 (14)	0.1940 (3)	0.8648 (3)	0.0386 (6)	0.50
H11S	0.5170	0.1448	0.9202	0.058*	0.50
H12S	0.5443	0.2473	0.8831	0.058*	0.50
C2S	0.5231 (3)	0.1445 (4)	0.7670 (3)	0.0762 (15)	0.50
H21S	0.5678	0.1498	0.7565	0.114*	0.50
H22S	0.5104	0.0738	0.7652	0.114*	0.50
C3S	0.4797 (3)	0.2050 (3)	0.6906 (4)	0.0720 (16)	0.50
H31S	0.5012	0.2656	0.6715	0.108*	0.50
H32S	0.4648	0.1657	0.6292	0.108*	0.50
C4S	0.4278 (2)	0.2296 (4)	0.7434 (3)	0.0701 (14)	0.50
H41S	0.4094	0.2944	0.7196	0.105*	0.50
H42S	0.3943	0.1786	0.7298	0.105*	0.50

### Atomic displacement parameters (Å^2^)

**Table:**

	*U*^11^	*U*^22^	*U*^33^	*U*^12^	*U*^13^	*U*^23^
P1	0.01646 (12)	0.01632 (11)	0.01603 (11)	0.00020 (9)	0.00027 (8)	−0.00027 (9)
O1	0.0221 (4)	0.0220 (4)	0.0238 (4)	0.0043 (3)	−0.0003 (3)	0.0014 (3)
O2	0.0340 (5)	0.0338 (5)	0.0348 (5)	−0.0149 (4)	0.0062 (4)	−0.0057 (4)
O3	0.0300 (4)	0.0246 (4)	0.0285 (4)	−0.0029 (3)	0.0075 (3)	0.0020 (3)
O4	0.0330 (5)	0.0477 (6)	0.0363 (5)	−0.0048 (4)	0.0103 (4)	−0.0206 (4)
C1	0.0174 (4)	0.0189 (4)	0.0169 (4)	−0.0015 (3)	0.0024 (3)	−0.0020 (3)
C2	0.0220 (5)	0.0203 (4)	0.0182 (4)	−0.0007 (4)	0.0032 (3)	0.0007 (4)
C3	0.0260 (5)	0.0209 (5)	0.0209 (4)	−0.0049 (4)	0.0050 (4)	−0.0006 (4)
C4	0.0196 (5)	0.0259 (5)	0.0183 (4)	−0.0045 (4)	0.0052 (3)	−0.0043 (4)
C5	0.0176 (5)	0.0259 (5)	0.0244 (5)	0.0008 (4)	0.0021 (4)	−0.0037 (4)
C6	0.0201 (5)	0.0197 (5)	0.0249 (5)	0.0004 (4)	0.0015 (4)	−0.0024 (4)
C7	0.0223 (5)	0.0335 (6)	0.0253 (5)	−0.0075 (4)	0.0059 (4)	−0.0076 (5)
C8	0.0164 (4)	0.0176 (4)	0.0172 (4)	0.0005 (3)	0.0021 (3)	−0.0003 (3)
C9	0.0196 (5)	0.0209 (4)	0.0166 (4)	−0.0014 (4)	0.0014 (3)	−0.0033 (3)
C10	0.0193 (4)	0.0190 (4)	0.0210 (4)	−0.0022 (4)	0.0035 (3)	−0.0027 (4)
C11	0.0202 (4)	0.0177 (4)	0.0190 (4)	0.0018 (3)	0.0049 (3)	−0.0010 (3)
C12	0.0251 (5)	0.0215 (5)	0.0162 (4)	−0.0001 (4)	0.0017 (4)	−0.0019 (3)
C13	0.0208 (5)	0.0186 (4)	0.0182 (4)	−0.0019 (4)	0.0008 (3)	−0.0025 (3)
C14	0.0263 (5)	0.0210 (5)	0.0206 (5)	0.0026 (4)	0.0076 (4)	−0.0006 (4)
C15	0.0177 (4)	0.0187 (4)	0.0159 (4)	−0.0024 (3)	0.0012 (3)	−0.0008 (3)
C16	0.0242 (5)	0.0221 (5)	0.0180 (4)	0.0012 (4)	0.0012 (4)	−0.0007 (4)
C17	0.0242 (5)	0.0250 (5)	0.0239 (5)	0.0005 (4)	0.0037 (4)	−0.0053 (4)
C18	0.0229 (5)	0.0287 (5)	0.0192 (4)	−0.0076 (4)	0.0053 (4)	−0.0064 (4)
C19	0.0231 (5)	0.0311 (5)	0.0160 (4)	−0.0066 (4)	0.0009 (4)	−0.0004 (4)
C20	0.0197 (4)	0.0230 (5)	0.0177 (4)	−0.0028 (4)	−0.0001 (3)	0.0013 (4)
C21	0.0289 (6)	0.0398 (7)	0.0235 (5)	−0.0105 (5)	0.0086 (4)	−0.0111 (5)
O1S	0.0330 (11)	0.0568 (14)	0.0539 (14)	−0.0007 (10)	−0.0072 (10)	0.0064 (11)
C1S	0.0297 (13)	0.0431 (17)	0.0399 (16)	−0.0004 (11)	−0.0043 (12)	−0.0067 (14)
C2S	0.107 (5)	0.078 (3)	0.039 (2)	0.021 (3)	0.001 (2)	−0.008 (2)
C3S	0.128 (5)	0.0307 (17)	0.046 (2)	−0.003 (2)	−0.022 (3)	0.0030 (17)
C4S	0.070 (3)	0.078 (3)	0.050 (2)	−0.030 (2)	−0.0275 (19)	0.036 (2)

### Geometric parameters (Å, º)

**Table:**

P1—O1	1.4880 (8)	C12—H12	0.9500
P1—C8	1.8050 (10)	C13—H13	0.9500
P1—C1	1.8083 (11)	C14—H14	0.9500
P1—C15	1.8129 (10)	C15—C20	1.3963 (14)
O2—C7	1.2099 (16)	C15—C16	1.4050 (15)
O3—C14	1.2107 (14)	C16—C17	1.3882 (15)
O4—C21	1.2078 (17)	C16—H16	0.9500
C1—C2	1.3975 (15)	C17—C18	1.3984 (16)
C1—C6	1.4009 (15)	C17—H17	0.9500
C2—C3	1.3905 (15)	C18—C19	1.3881 (17)
C2—H2	0.9500	C18—C21	1.4830 (16)
C3—C4	1.3968 (16)	C19—C20	1.3944 (15)
C3—H3	0.9500	C19—H19	0.9500
C4—C5	1.3924 (16)	C20—H20	0.9500
C4—C7	1.4851 (15)	C21—H21	0.9500
C5—C6	1.3895 (15)	O1S—C4S	1.418 (4)
C5—H5	0.9500	O1S—C1S	1.422 (4)
C6—H6	0.9500	C1S—C2S	1.519 (5)
C7—H7	0.9500	C1S—H11S	0.9900
C8—C13	1.3981 (14)	C1S—H12S	0.9900
C8—C9	1.4049 (14)	C2S—C3S	1.510 (6)
C9—C10	1.3873 (15)	C2S—H21S	0.9900
C9—H9	0.9500	C2S—H22S	0.9900
C10—C11	1.4013 (14)	C3S—C4S	1.446 (8)
C10—H10	0.9500	C3S—H31S	0.9900
C11—C12	1.3909 (15)	C3S—H32S	0.9900
C11—C14	1.4839 (15)	C4S—H41S	0.9900
C12—C13	1.3904 (15)	C4S—H42S	0.9900
			
O1—P1—C8	113.74 (5)	C11—C14—H14	117.6
O1—P1—C1	112.73 (5)	C20—C15—C16	120.01 (10)
C8—P1—C1	106.20 (5)	C20—C15—P1	116.88 (8)
O1—P1—C15	111.61 (5)	C16—C15—P1	123.09 (8)
C8—P1—C15	106.89 (5)	C17—C16—C15	119.67 (10)
C1—P1—C15	105.07 (5)	C17—C16—H16	120.2
C2—C1—C6	119.95 (10)	C15—C16—H16	120.2
C2—C1—P1	118.12 (8)	C16—C17—C18	120.06 (11)
C6—C1—P1	121.87 (8)	C16—C17—H17	120.0
C3—C2—C1	120.11 (10)	C18—C17—H17	120.0
C3—C2—H2	119.9	C19—C18—C17	120.31 (10)
C1—C2—H2	119.9	C19—C18—C21	119.37 (11)
C2—C3—C4	119.68 (10)	C17—C18—C21	120.29 (11)
C2—C3—H3	120.2	C18—C19—C20	119.97 (10)
C4—C3—H3	120.2	C18—C19—H19	120.0
C5—C4—C3	120.36 (10)	C20—C19—H19	120.0
C5—C4—C7	118.73 (11)	C19—C20—C15	119.93 (10)
C3—C4—C7	120.91 (10)	C19—C20—H20	120.0
C6—C5—C4	120.07 (10)	C15—C20—H20	120.0
C6—C5—H5	120.0	O4—C21—C18	124.90 (12)
C4—C5—H5	120.0	O4—C21—H21	117.6
C5—C6—C1	119.79 (10)	C18—C21—H21	117.6
C5—C6—H6	120.1	C4S—O1S—C1S	107.6 (3)
C1—C6—H6	120.1	O1S—C1S—C2S	107.0 (3)
O2—C7—C4	124.11 (12)	O1S—C1S—H11S	110.3
O2—C7—H7	117.9	C2S—C1S—H11S	110.3
C4—C7—H7	117.9	O1S—C1S—H12S	110.3
C13—C8—C9	120.03 (9)	C2S—C1S—H12S	110.3
C13—C8—P1	120.75 (8)	H11S—C1S—H12S	108.6
C9—C8—P1	119.01 (7)	C3S—C2S—C1S	101.2 (4)
C10—C9—C8	120.31 (9)	C3S—C2S—H21S	111.5
C10—C9—H9	119.8	C1S—C2S—H21S	111.5
C8—C9—H9	119.8	C3S—C2S—H22S	111.5
C9—C10—C11	119.38 (10)	C1S—C2S—H22S	111.5
C9—C10—H10	120.3	H21S—C2S—H22S	109.4
C11—C10—H10	120.3	C4S—C3S—C2S	103.1 (4)
C12—C11—C10	120.30 (10)	C4S—C3S—H31S	111.2
C12—C11—C14	118.26 (9)	C2S—C3S—H31S	111.2
C10—C11—C14	121.44 (10)	C4S—C3S—H32S	111.2
C13—C12—C11	120.57 (9)	C2S—C3S—H32S	111.2
C13—C12—H12	119.7	H31S—C3S—H32S	109.1
C11—C12—H12	119.7	O1S—C4S—C3S	109.3 (3)
C12—C13—C8	119.39 (10)	O1S—C4S—H41S	109.8
C12—C13—H13	120.3	C3S—C4S—H41S	109.8
C8—C13—H13	120.3	O1S—C4S—H42S	109.8
O3—C14—C11	124.87 (10)	C3S—C4S—H42S	109.8
O3—C14—H14	117.6	H41S—C4S—H42S	108.3
			
O1—P1—C1—C2	−8.16 (10)	C14—C11—C12—C13	178.05 (10)
C8—P1—C1—C2	−133.35 (8)	C11—C12—C13—C8	0.44 (16)
C15—P1—C1—C2	113.60 (9)	C9—C8—C13—C12	1.05 (16)
O1—P1—C1—C6	174.63 (8)	P1—C8—C13—C12	175.71 (8)
C8—P1—C1—C6	49.43 (10)	C12—C11—C14—O3	179.01 (11)
C15—P1—C1—C6	−63.62 (10)	C10—C11—C14—O3	−1.64 (17)
C6—C1—C2—C3	1.99 (16)	O1—P1—C15—C20	11.07 (10)
P1—C1—C2—C3	−175.28 (8)	C8—P1—C15—C20	136.02 (8)
C1—C2—C3—C4	−0.51 (15)	C1—P1—C15—C20	−111.41 (9)
C2—C3—C4—C5	−1.42 (16)	O1—P1—C15—C16	−170.73 (9)
C2—C3—C4—C7	178.15 (10)	C8—P1—C15—C16	−45.78 (10)
C3—C4—C5—C6	1.87 (16)	C1—P1—C15—C16	66.79 (10)
C7—C4—C5—C6	−177.70 (10)	C20—C15—C16—C17	0.10 (16)
C4—C5—C6—C1	−0.39 (16)	P1—C15—C16—C17	−178.04 (8)
C2—C1—C6—C5	−1.54 (16)	C15—C16—C17—C18	−1.91 (17)
P1—C1—C6—C5	175.63 (8)	C16—C17—C18—C19	2.16 (17)
C5—C4—C7—O2	175.74 (11)	C16—C17—C18—C21	−175.80 (11)
C3—C4—C7—O2	−3.83 (17)	C17—C18—C19—C20	−0.57 (17)
O1—P1—C8—C13	−97.60 (9)	C21—C18—C19—C20	177.40 (10)
C1—P1—C8—C13	26.97 (10)	C18—C19—C20—C15	−1.24 (16)
C15—P1—C8—C13	138.76 (9)	C16—C15—C20—C19	1.48 (16)
O1—P1—C8—C9	77.11 (9)	P1—C15—C20—C19	179.73 (8)
C1—P1—C8—C9	−158.32 (8)	C19—C18—C21—O4	177.78 (12)
C15—P1—C8—C9	−46.53 (9)	C17—C18—C21—O4	−4.24 (19)
C13—C8—C9—C10	−1.68 (16)	C4S—O1S—C1S—C2S	−11.3 (4)
P1—C8—C9—C10	−176.43 (8)	O1S—C1S—C2S—C3S	28.0 (4)
C8—C9—C10—C11	0.82 (16)	C1S—C2S—C3S—C4S	−33.6 (5)
C9—C10—C11—C12	0.67 (16)	C1S—O1S—C4S—C3S	−11.4 (4)
C9—C10—C11—C14	−178.66 (10)	C2S—C3S—C4S—O1S	29.0 (5)
C10—C11—C12—C13	−1.31 (16)		

### Hydrogen-bond geometry (Å, º)

**Table:**

*D*—H···*A*	*D*—H	H···*A*	*D*···*A*	*D*—H···*A*
C7—H7···O3^i^	0.95	2.56	3.4303 (16)	152
C14—H14···O1^ii^	0.95	2.50	3.1575 (14)	127

Symmetry codes: (i) *x*+1/2, *y*+1/2, *z*; (ii) −*x*+1/2, −*y*+1/2, −*z*+1.
